# Association of NRAS Mutation With Clinical Outcomes of Anti-PD-1 Monotherapy in Advanced Melanoma: A Pooled Analysis of Four Asian Clinical Trials

**DOI:** 10.3389/fimmu.2021.691032

**Published:** 2021-07-05

**Authors:** Li Zhou, Xuan Wang, Zhihong Chi, Xinan Sheng, Yan Kong, Lili Mao, Bin Lian, Bixia Tang, Xieqiao Yan, Xue Bai, Siming Li, Jun Guo, Chuanliang Cui, Lu Si

**Affiliations:** Key Laboratory of Carcinogenesis and Translational Research (Ministry of Education/Beijing), Department of Renal Cancer and Melanoma, Peking University Cancer Hospital & Institute, Beijing, China

**Keywords:** NRAS, noncutaneous melanoma, cutaneous melanoma, immunotherapy, anti-PD-1 monotherapy

## Abstract

**Background:**

Anti-PD-1 monotherapy is the standard therapy for advanced melanoma patients, including those with NRAS mutations. The influence of NRAS mutation on immunotherapy, especially in noncutaneous melanoma, is largely uncharacterized.

**Materials and Methods:**

We analyzed clinical data of four clinical trials for advanced melanoma patients treated with anti-PD-1 monotherapy between 2016 and 2019. The impact of NRAS mutation on efficacy and outcome of immunotherapy were analyzed in cutaneous and noncutaneous groups separately.

**Results:**

A total of 206 patients were assessed, including 92 cutaneous melanoma patients with 12 NRAS mutations and 114 noncutaneous melanoma patients with 21 NRAS mutations. In cutaneous melanoma, the response rates of NRAS mutant patients were lower than patients without NRAS mutations (9.5% *vs*. 23.9%), the median progression-free survival (PFS) and median overall survival (OS) were shorter for patients with NRAS mutations, although without significant difference for OS (P=0.081). In noncutaneous melanoma, the response rates were 0 and 13.7% for NRAS mutant and wild-type patients, the median PFS were 3.6 months (95% CI: 0.9-6.3) and 4.3 months (95%CI: 2.9-5.7) (P=0.015), and the median OS were 10.8 months (95% CI: 1.5-20.1) and 15.3 months (95% CI: 13.2-17.4) (P=0.025), respectively. In multivariate analysis, NRAS mutation, along with ECOG performance score and LDH level, was negatively associated with both PFS (HR 1.912, P=0.044) and OS (HR 2.210, P=0.025) in noncutaneous melanoma.

**Conclusion:**

In advanced Asian melanoma treated with anti-PD-1 monotherapy, NRAS mutant patients had lower response rates and poorer prognoses compared to wild-type patients, especially in noncutaneous subtypes.

## Introduction

Cutaneous melanomas are classified into four genetic subtypes based on TCGA (The Cancer Genome Atlas): BRAF mutant, RAS mutant, NF1 mutant, and triple WT (wild-type) (1). The incidence of BRAF mutation is 35-50% in cutaneous melanoma, while the upstream NRAS mutation frequency is 15-20%. BRAF inhibitors in combination with MEK inhibitors are recommended as the standard treatment for BRAF mutant advanced melanoma (2). However, for NRAS mutant melanoma, MEK inhibitors and other targeted therapies are still under investigation. Binimetinib slightly improved progression-free survival (PFS) of NRAS mutant melanoma patients by 1.3 months compared to dacarbazine (2.8 *vs*. 1.5 months) in a phase III clinical trial (3). Immunotherapy is still the first-line recommendation for advanced NRAS mutant melanoma.

Melanoma patients from different ethnicities have distinct subtypes and genomic alterations. Instead of the predominant cutaneous subtype in Caucasians, acral melanoma and mucosal melanoma are the most common subtypes in non-Caucasians (4). Genomic differences are substantial across different races and subtypes. For example, KIT aberrations are more common in acral and mucosal melanoma (5). The frequencies of BRAF mutation and NRAS mutation are 23.7% and 10.4%, respectively, based on calculations in a large non-Caucasian population (6). The incidences of NRAS mutations in acral, mucosal, and cutaneous melanoma were 9.0%, 13.0%, and 10.8% respectively (6). Acral and mucosal subtypes were less responsive to immunotherapy than cutaneous melanoma in a series of studies (7–9) due to low tumor mutation burden and high proportion of copy number variations and chromosomal structure variations (10).

Some retrospective studies have investigated the response and outcome of immunotherapy for NRAS mutant melanoma. Studies of high-dose interleukin-2 (IL-2) and ipilimumab have reported increased objective response rates (ORRs) and improved overall survival (OS) in patients with NRAS mutations, although without significant differences (11, 12). Johnson et al. (13) showed the benefit of immunotherapy in advanced melanoma patients with NRAS mutation exceeded that in patients with wild-type melanoma, especially from anti-PD-1/PD-L1 antibodies (ORR 64% *vs*. 30%). In contrast, in a German analysis, immune checkpoint inhibitors yielded comparable response rates (21% *vs*. 13%, P=0.210) but inferior survival in NRAS mutant melanoma (14). Patients in these studies mainly had melanoma from cutaneous primary sites. However, the influence of NRAS mutation on the efficacy of immunotherapy in noncutaneous melanoma has not been extensively explored.

Therefore, we conducted this observational study by collecting information on patients with advanced NRAS mutant melanoma and wild-type patients who received immunotherapy. By analyzing the different responses to immunotherapy in patients with NRAS mutations between cutaneous and noncutaneous melanoma, we analyzed the association of NRAS mutation with immunotherapy outcome in Asian melanoma population and tried to identify potential treatment strategies for these patients.

## Materials and Methods

We collected clinical data of advanced melanoma patients treated with anti-PD-1 monotherapy from four prospective clinical trials conducted in Peking University Cancer Hospital (NCT03013101, NCT02821000, NCT02738489, CTR20160872). The impact of NRAS mutation on efficacy and outcome of immunotherapy for advanced melanoma patients was analyzed among other clinical characteristics. This study was approved by the ethics committee of the Peking University Cancer Hospital. NRAS, BRAF, and KIT mutations were detected by PCR from formalin-fixed paraffin-embedded (FFPE) tissue. In this study, NRAS wild-type melanoma included patients with BRAF or KIT mutations.

All patients received anti-PD-1 antibody, including pembrolizumab, toripalimab, tislelizumab, and camrelizumab, as a systemic treatment for advanced disease in clinical trials. Toripalimab had an ORR of 17.3% in previously treated melanoma (7), with tislelizumab 15% (15) and camrelizumab 15.2% (yet to be published), which was consistent with 16.7% for pembrolizumab in a second-line setting of Keynote 151 trial (9) in Asian melanoma patients. Nivolumab was not involved because it had not been involved in clinical trials tested for melanoma in China. An ECOG (Eastern Cooperative Oncology Group) performance score of 0 or 1 was required. Prior chemotherapy or targeted therapy was allowed. Patients with no detailed demographic information or missing efficacy evaluation data and those unable to complete the treatment cycle for any reason were excluded.

Responses were evaluated using the Response Evaluation Criteria in Solid Tumors (RECIST) version 1.1 and Immune-related Response Evaluation Criteria in Solid Tumors (irRECIST). Patients were allowed to continue on treatment when initially progressed per RECIST v1.1 but may benefit from the continuation of immunotherapy. Treatment continued until intolerable toxicities developed, a complete response (CR) was achieved, or a 2-year treatment course was completed. The objective of this study was to explore the impact of NRAS mutation on ORR, disease control rate (DCR), PFS, and OS in advanced melanoma patients who received anti-PD-1 monotherapy.

Given the distinct genomic alterations and response to immunotherapy between different subtypes, we divided the patient population into a cutaneous cohort and a noncutaneous cohort and analyzed these two cohorts separately. The cutaneous cohort included patients with melanoma arising from skin and unknown primary sites. Acral melanoma is one special subtype distinct from non-acral cutaneous melanoma. Therefore, we categorized acral and mucosal melanoma into the noncutaneous cohort.

Chi-square tests or Fisher’s exact tests were used for categorical data. Survival data were analyzed with the Kaplan-Meier method, and log-rank tests were used for comparisons between different groups. Cox proportional hazards regression analysis was conducted to identify the following possible predictors of PFS and OS in advanced noncutaneous melanoma patients: NRAS status (wild-type *vs*. mutant), BRAF status (wild-type *vs*. mutant), ECOG performance score (0 *vs*. 1), primary site (acral *vs*. mucosal origins), stage (advanced IIIc *vs*. IV; based on the American Joint Committee on Cancer (AJCC) 8^th^ edition staging system for cutaneous melanoma), and serum lactic dehydrogenase (LDH) level (normal *vs*. elevated). Statistical analyses were performed using SPSS software (version 22.0; IBM Corporation, Armonk, NY) and GraphPad PRISM (Prism 8.0.2; GraphPad Software, LLC). A two-sided P value <0.05 was considered statistically significant.

We performed a systematic search of relevant prospective and retrospective studies in MEDLINE limited to the English language on June 5, 2021. We included all studies that compared the efficiency of immunotherapy in NRAS mutant with NRAS wild-type advanced melanoma patients. We calculated weighted event rates and 95% CIs by using Stata version 16.0 software. A random rather than fixed-effects model was used to estimate pooled event rates to account for heterogeneity in order to be more conservative. We evaluated heterogeneity between studies with the Higgins inconsistency index (*I* ^2^).

## Results

### Baseline Characteristics

A total of 206 advanced melanoma patients were enrolled in this study, including 92 cutaneous melanoma patients with 12 NRAS mutations and 114 noncutaneous melanoma patients with 21 NRAS mutations. The detailed characteristics are listed in **Table 1**. The distribution of sex, age, ECOG performance score, stage, serum LDH level, and prior therapy was balanced between the NRAS mutant and wild-type patients in both the cutaneous and noncutaneous groups. All patients were immunotherapy-naïve, and 170 patients (82.5%) had previously received chemotherapy or targeted therapy. There were 75 acral melanoma and 39 mucosal melanoma patients in the noncutaneous cohort.

**Table 1:** Patient characteristics.

Characteristics	Cutaneous N=92			Noncutaneous N=114			
	NRAS Mutant N=21	NRAS Wild-type N=71	P value	NRAS Mutant N=12		NRAS Wild-type N=102	P value
**Sex**			0.948				0.758
** Female**	12 (57.1)	40 (56.3)		8 (66.7)		59 (57.8)	
** Male**	9 (42.9)	31 (43.7)		4 (33.3)		43 (42.2)	
**Age, years**			0.164				1.000
** <65**	16 (76.2)	63 (88.7)		10 (83.3)		86 (84.3)	
** >=65**	5 (23.8)	8 (11.3)		2 (16.7)		16 (15.7)	
**ECOG PS**			0.685				0.365
** 0**	12 (57.1)	37 (52.1)		3 (25.0)		41 (40.2)	
** 1**	9 (42.9)	34 (47.9)		9 (75.0)		61 (59.8)	
**Stage**			0.377				0.356
** IIIc**	3 (14.3)	5 (7.0)		0 (0)		14 (13.7)	
** IV**	18 (85.7)	66 (93.0)		12 (100)		88 (86.3)	
**LDH level**			0.161				0.209
** Normal**	18 (85.7)	50 (70.4)		5 (41.7)		65 (63.7)	
** Elevated**	3 (14.3)	21 (29.6)		7 (58.3)		37 (36.3)	
**Prior therapy**			0.127				0.384
** Naïve**	7 (33.3)	12 (16.9)		3 (25.0)		14 (13.7)	
** Treated**	14 (66.7)	59 (83.1)		9 (75.0)		88 (86.3)	
**BRAF mutation**	0	24 (33.8)	**0.002**	0		9 (8.8)	0.594
**KIT mutation**	0	3 (4.2)	1.000	1 (8.3)		9 (8.8)	1.000

ECOG PS, Eastern Cooperative Oncology Group performance score; LDH, lactic dehydrogenase. P values < 0.05 were in bold.

BRAF and NRAS mutations were mutually exclusive, and the BRAF mutation frequency was 19.1% in the NRAS wild-type population in our cohort. A total of 15 patients with BRAF mutation received BRAF inhibitors with or without MEK inhibitors before or after immunotherapy. Only one patient harbored simultaneous NRAS and KIT mutations. Twelve patients had KIT alterations in the NRAS wild-type cohort. Q61 mutations were predominant among NRAS mutation hotspots, including Q61R (52%, n=17), Q61K (24%, n=8), Q61L (9%, n=3), and Q61H (3%, n=1). Other mutational hotspots included G12D (9%, n=3) and G12C (3%, n=1).

### Efficacy Evaluation

In cutaneous melanoma, 21 from 92 patients had NRAS mutations. The overall ORR of anti-PD-1 monotherapy in the NRAS mutant population was 9.5%, which was lower than the rate of 23.9% among the wild-type patients (P=0.223). The DCRs were 47.6% in the NRAS mutant group and 66.2% in the wild-type group (P=0.123). In the noncutaneous melanoma population, 12 from 114 patients had NRAS mutations, including 9 acral and 3 mucosal melanoma patients. The response rates of NRAS mutant patients and NRAS wild-type patients were 0% and 13.7% (P=0.356), and the DCRs were 33.3% and 51.0% (P=0.247), respectively (**Table 2**).

**Table 2:** Efficacy of immunotherapy in NRAS mutant and NRAS wild-type melanoma.

	NRAS Mutant N=33 (%)	NRAS Wild-type N=173 (%)	P value
Cutaneous*	N=21	N=71	
**ORR**	2(9.5)	17(23.9)	0.223
**DCR**	10(47.6)	47(66.2)	0.123
**Noncutaneous**	N=12	N=102	
**ORR**	**0(0)**	14(13.7)	0.356
**DCR**	4(33.3)	52(51.0)	0.247
** Acral**	N=9	N=66	
**ORR**	**0(0)**	8(12.1)	0.585
**DCR**	4(44.4)	36(54.5)	0.726
** Mucosal**	N=3	N=36	
**ORR**	**0(0)**	6(16.7)	1.000
**DCR**	0(0)	16(44.4)	0.255

*Melanoma of unknown primary site included. Subgroups with no responses were in bold.

Among the patients with NRAS mutation receiving immunotherapy, the only two responsive patients were both in the cutaneous melanoma group (9.5%). No responsive cases were observed in the noncutaneous group. No NRAS mutant patients had BRAF mutations simultaneously, and one patient with NRAS and KIT aberrations at the same time experienced stable disease (SD) in response to immunotherapy.

Among the patients without NRAS mutation, the ORRs of anti-PD-1 monotherapy were 23.9% for cutaneous melanoma and 13.7% for noncutaneous melanoma as follows: 12.1% in acral melanoma and 16.7% in mucosal melanoma. This population consisted of patients with BRAF mutation and KIT aberration. Patients with BRAF mutation had a slightly better ORR than BRAF/NRAS wild-type patients (21.1% *vs*. 17.1%). Patients with KIT aberration had an ORR of 8.3% (1/12) to immunotherapy.

Two patients with NRAS mutant advanced melanoma, including 1 patient with amino acid mutation of Q61R and 1 patient with G12D, achieved a partial response (PR). Regarding mutational hotspots with frequencies greater than 10%, patients with Q61R had a better ORR (5.8% *vs*. 0) and DCR (53% *vs*. 25%) than those with Q61K. One patient with Q61H and one with G12C had SD, and all the other patients experienced progressive disease (PD) (**Table 3**).

**Table 3:** Efficacy of immunotherapy by NRAS mutational hotspot.

Hotspot	Q61R	Q61K	Q61L	Q61H	G12D	G12C
**N (%)**	17(52%)	8(24%)	3(9%)	1(3%)	3(9%)	1(3%)
**PR**	1	0	0	0	1	0
**SD**	8	2	1	0	0	1
**PD**	8	6	2	1	2	0

### Survival Analysis

**Figure 1:**
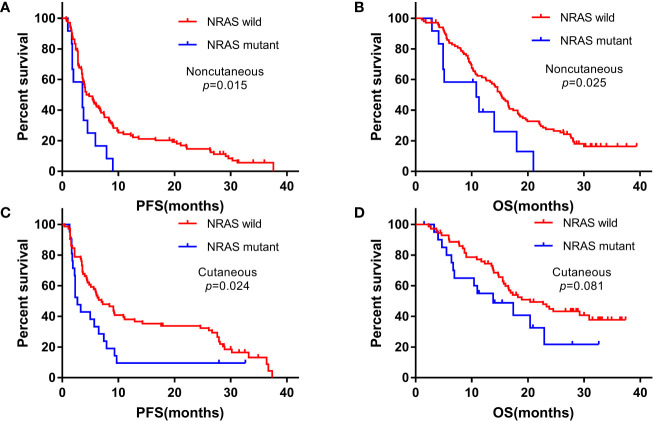
Kaplan-Meier curves for progression-free survival (PFS) and overall survival (OS) among patients with NRAS mutant and wild-type statuses treated with anti-PD-1 monotherapy. **(A, B)** noncutaneous melanoma; **(C, D)** cutaneous melanoma.

We analyzed the association of NRAS mutations with survival in cutaneous and noncutaneous melanoma. PFS and OS were poorer in patients with NRAS mutation, especially in the noncutaneous group. With a median follow-up duration of 15.4 months (range: 1.1-39.4), the Kaplan-Meier curves of PFS and OS for advanced NRAS mutant melanoma patients are shown in **Figure 1**.

In the cutaneous melanoma cohort, the PFS for NRAS mutant and wild-type patients were 2.7 months (95%CI: 1.7-3.7) and 7.0 months (95%CI: 4.1-9.9) (P=0.024), and the OS were 13.8 months (95%CI: 3.7-23.9) and 20.4 months (95%CI: 12.7-28.1) (P=0.081), respectively. In the noncutaneous melanoma cohort, the median PFS were 3.6 months (95% CI: 0.9-6.3) for NRAS mutant patients and 4.3 months (95%CI: 2.9-5.7) for wild-type patients (P=0.015), respectively, and the median OS were 10.8 months (95% CI: 1.5-20.1) and 15.3 months (95% CI: 13.2-17.4), respectively (P=0.025).

We further explored the role of NRAS mutation in noncutaneous melanoma. In a univariate analysis incorporating all factors, the ECOG performance score (P=0.004), LDH level (P<0.001), and NRAS mutation status (P=0.015) were significantly associated with PFS in noncutaneous melanoma. In addition, the ECOG performance score (P<0.001), LDH level (P<0.001), and NRAS mutation (P=0.025) were also significantly associated with OS. On multivariate analysis, covariates independently associated with improved PFS included an ECOG performance score of 0, a normal LDH level, and NRAS wild-type status. Predictive factors for OS identified from the Cox hazard ratio model also included ECOG performance score, LDH level, and NRAS mutation status, as shown in **Table 4**.

**Table 4:** Multivariate analysis of factors for PFS and OS in patients with advanced noncutaneous melanoma receiving immunotherapy.

	**PFS**			**OS**		
	HR	95% CI	P value	HR	95% CI	P value
**NRAS**						
**Wild-type**	Ref			Ref		
**Mutant**	1.912	1.017-3.592	**0.044**	2.210	1.105-4.420	**0.025**
**BRAF**						
**Wild-type**	Ref			Ref		
**Mutant**	0.722	0.331-1.577	0.414	0.907	0.414-1.990	0.808
**ECOG PS**						
**0**	Ref			Ref		
**1**	1.530	1.003-2.335	**0.048**	1.934	1.191-3.141	**0.008**
**Primary site**						
**Acral**	Ref			Ref		
**Mucosal**	0.989	0.641-1.525	0.959	1.106	0.699-1.751	0.666
**Stage**						
**IIIc**	Ref			Ref		
**IV**	0.987	0.552-1.765	0.964	0.853	0.432-1.683	0.646
**LDH level**						
**Normal**	Ref			Ref		
**Elevated**	1.992	1.321-3.005	**0.001**	2.234	1.430-3.488	**<0.001**

PFS, progression-free survival; OS, overall survival; HR, hazard ratio; 95% CI, 95% confidence interval; Ref, reference; ECOG PS, Eastern Cooperative Oncology Group performance score. P values < 0.05 were in bold.

### Meta-Analysis

Six retrospective studies and 1 randomized clinical trial were relevant to our analyses (11–14, 16–18). Study design, drug, number of patients, efficacy of immunotherapy (ORR, DCR, PFS, OS, TTF, et al) for NRAS mutant and wild-type advanced melanoma patients, and univariate and multivariate factors analyzed along with NRAS status were listed in **Supplementary Table S1**. The objectives of our study were ORR, PFS, and OS. However, these studies were highly heterogenous and not consistent in the objectives. Only part of the studies can be involved for different pooled analyses.

**Figure 2:**
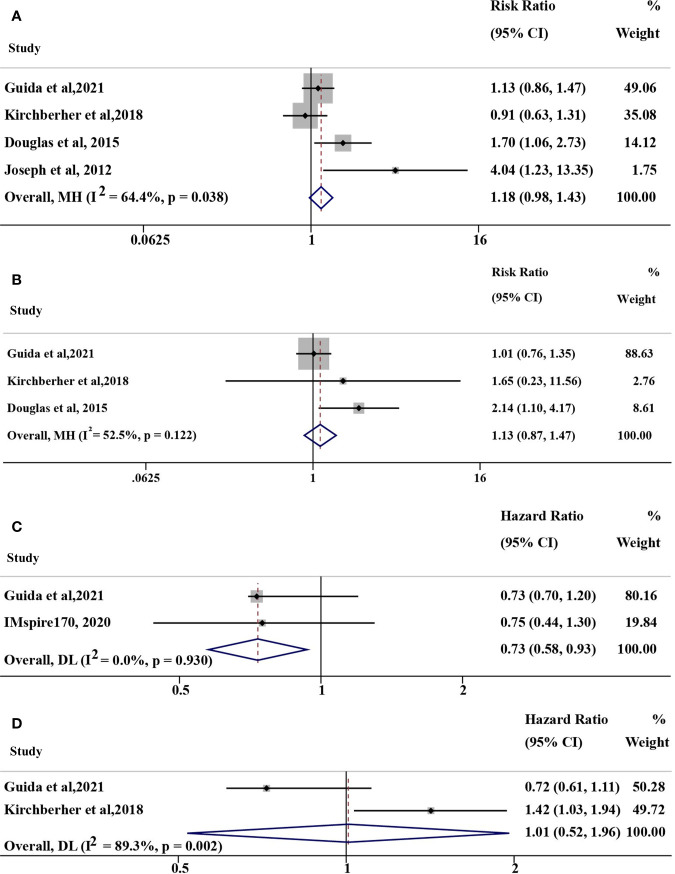
Overall pooled analyses of efficacy for NRAS mutant *vs*. wild-type (mut/wt) advanced melanoma treated with immunotherapy, including IL-2, anti-PD-(L)1, anti-CTLA-4, and anti-PD-1+anti-CTLA-4 therapy. **(A)** Risk ration (RR) of ORR for NRAS mut/wt melanoma treated with immunotherapy; **(B)** RR of ORR for NRAS mut/wt melanoma treated with anti-PD-1 monotherapy; **(C)** Hazard ratio (HR) of PFS for NRAS mut/wt melanoma treated with immunotherapy; **(D)** HR of OS for NRAS mut/wt melanoma treated with immunotherapy. *I* ^2^ was interpreted by <50% as low heterogeneity; 50% to 75%, moderate heterogeneity; and >75% as high heterogeneity in our study.

The pooled risk ratio (RR) of ORR was 1.18 (95% CI: 0.98-1.43, *I* ^2^ = 64.4%, P=0.038; **Figure 2A**) for some of the above studies using immunotherapy (including anti-PD-(L)1, anti-CTLA-4, anti-PD-1+anti-CTLA-4 therapy and IL-2). In the anti-PD(L)1 monotherapy subgroup, the corresponding RR of ORR was 1.13 (95% CI: 0.87-1.47, *I* ^2^ = 52.5%, P=0.122; **Figure 2B**) with moderate heterogeneity. The hazard ratio (HR) of PFS in 2 studies (16, 17) and overall survival (OS) in 2 studies (14, 17) were 0.73 (95% CI: 0.58-0.93, *I* ^2^ = 0%, P=0.930; **Figure 2C**) and 1.01 (95% CI: 0.52-1.96, *I* ^2^ = 89.3%, P=0.002; **Figure 2D**), respectively. However, studies with opposite results were not included because of different objectives, such as TTF (18). As a result, no confirmatory conclusion can be drawn from this meta-analysis, which demonstrated the controversial results of different published studies.

## Discussion

To our knowledge, this study of immunotherapy for advanced NRAS mutant melanoma comprised the largest population of noncutaneous melanoma to date. Currently, only a few reports have addressed the impact of NRAS mutation on immunotherapy. In an analysis of the effects of mutations on the response to high-dose IL-2, patients with NRAS mutation demonstrated a higher ORR (47%) than those with BRAF mutation (23%) and wild-type status (12%) (P=0.05) (11). In addition, NRAS mutation had an association with improved OS in ipilimumab-treated melanoma compared with wild-type melanoma, although without a significant difference (12 *vs*. 8 months, P=0.56) (12). In a retrospective study, 11 patients with NRAS mutant melanoma and 37 wild-type patients received anti-PD-1 monotherapy. The ORRs were 64% and 30% in the NRAS mutant and wild-type groups, respectively (13). Another study suggested that response rates were comparable between the groups (21% *vs*. 13%, P=0.210), although NRAS mutation was associated with less favorable survival (14). However, results from an Asian multicenter phase II trial of toripalimab in advanced melanoma patients indicated NRAS mutation as a potential resistance mechanism for immunotherapy. In the Asian population predominantly with acral and mucosal melanoma, the ORR of NRAS mutant patients was only 6.1% (1/16) to anti-PD-1 monotherapy (7). In a recent study of MAPK pathway alteration in cutaneous and unknown primary melanomas, time to treatment failure (TTF) was shorter for patients with NRAS Q61 mutations (18).

We performed a systemic meta-analysis based on the relevant studies. However, no confirmatory conclusion had been reached due to the high heterogeneity and no consistent objectives of these studies. The patients involved in different studies varied a lot in baseline characteristics. Only two studies involved a small part of acral and mucosal melanoma. In general, no consensus has been reached on the impact of NRAS mutation on immunotherapy, especially in noncutaneous melanoma.

In our study, the ORRs and DCRs of NRAS mutant melanoma patients were lower than those of wild-type patients, and NRAS mutation was associated with worse survival in the noncutaneous group with a significant difference. Moreover, no responsive patients were noted in the noncutaneous group with NRAS mutations. Advanced melanoma patients from Asia were less responsive to immunotherapy based on previous clinical trials, even in cutaneous melanoma, perhaps due to different races (7, 9). Noncutaneous melanoma is distinct from cutaneous melanoma in terms of subtypes and genetic alterations. According to whole-genome sequencing results, acral and mucosal subtypes were dominated by structural and copy number variations instead of single nucleotide variations (10). Genetic aberrations in the CDK and PI3K/AKT/mTOR pathways are frequently detected (19–21). All of these factors may contribute to the relatively low response rates of immunotherapy in our cohorts. Moreover, the particularly low response rate of noncutaneous melanoma patients with NRAS mutation reveals the adverse impact of NRAS mutation on immunotherapy in the Asian population. Our finding suggests that NRAS mutation might play a negative role in ethnic groups with deeper skin colors and a low tumor mutation burden.

MAPK pathway activation is associated with a poor prognosis in metastatic melanoma (22). NRAS mutant melanoma has been demonstrated to be associated with more unfavorable survival than wild-type melanoma in some studies, although heterogeneous situations have been observed in other series (23, 24). Increasing numbers of studies have explored how somatic alterations influence the response of immunotherapy through immunogenicity and the immune microenvironment (25). Tumors with NRAS mutation are reported to have low tumor-infiltrating lymphocyte (TIL) grades, suggesting a more immunosuppressive microenvironment (26). Activation of the RAS pathway can decrease antigen-presenting major histocompatibility complex (MHC) I molecule expression and reduce the number of infiltrating immune cells in tumors (24), which may weaken the antitumor activity of immune checkpoint inhibitors. This observation is consistent with our result of a significantly worse prognosis in NRAS mutant noncutaneous melanoma. A comprehensive understanding of the mechanisms by which somatic mutations influence the immune landscape and molecular network in the tumor microenvironment is critical to clarify this problem.

Thus, anti-PD-1 monotherapy may not be enough for patients with NRAS mutant advanced melanoma, especially in noncutaneous subtypes. NRAS is the most undruggable target in melanoma. MEK, PI3K, RalGEF, and other downstream molecules in the complex network have been targeted, with modest effects. On the other hand, considerable exploration of combination therapy in NRAS mutant melanoma has been conducted. MEK inhibitor treatment combined with immunotherapy was previously the most promising strategy. However, the role of MEK inhibitors in immunotherapy is controversial. MEK inhibition not only resulted in an accumulation of intratumoral antigen-specific T cells but also impaired T cell priming in lymph nodes (27). Preclinical evidence shows synergistic antitumor activity of MEK inhibition in combination with PD-L1 checkpoint blockade (27). In contrast, the phase 3 clinical trial of atezolizumab combined with cobimetinib in metastatic melanoma failed to demonstrate superior survival over anti-PD-1 monotherapy (16). Further studies are focusing on the sequence of immunotherapy and targeted therapy and dosing schedules such as intermittent versus continuous dosing of MAPK inhibitors (28). Other combination strategies, including drugs targeting RAS, the PI3K/AKT/mTOR pathway, CDK, and alternative immune checkpoint inhibitors, are also being investigated.

Some limitations exist in our study. This was an *ad hoc* analysis of pooled data from four clinical trials. Due to the unavailability of ipilimumab, we enrolled only patients with NRAS mutant advanced melanoma receiving anti-PD-1 monotherapy. As anti-PD-1 antibodies were tested mainly in previously treated melanoma in China, most patients received anti-PD-1 monotherapy after chemotherapy. Prospective multicenter study of large sample is needed to confirm the role of NRAS mutation in the response to immunotherapy in patients with advanced melanoma.

## Conclusion

Our study demonstrated that patients with advanced NRAS mutant melanoma had lower response rates and worse prognoses when treated with anti-PD-1 monotherapy than wild-type patients. New approaches are needed to improve the outcomes of NRAS mutant melanoma, especially in noncutaneous melanoma.

## Data Availability Statement

The raw data supporting the conclusions of this article will be made available by the authors, without undue reservation.

## Ethics Statement

The studies involving human participants were reviewed and approved by The ethics committee of the Peking University Cancer Hospital. The patients/participants provided their written informed consent to participate in this study.

## Author Contributions

LS, LZ, CC, JG, and XW designed the study. LZ and XW analyzed the data and wrote the manuscript. ZC, XS, YK, LM, BL, BT, XY, XB, and SL collected the data. All authors contributed to the article and approved the submitted version.

## Funding

This work was supported by grant no. 81972566 from the National Natural Science Foundation of China.

## Conflict of Interest

The authors declare that the research was conducted in the absence of any commercial or financial relationships that could be construed as a potential conflict of interest.
