# Supplementary material for: Association of NRAS Mutation With Clinical Outcomes of Anti-PD-1 Monotherapy in Advanced Melanoma: A Pooled Analysis of Four Asian Clinical Trials
Source: Front Immunol. 2021 Jul 5;12:691032. doi: 10.3389/fimmu.2021.691032 (PMC8289467; doi:10.3389/fimmu.2021.691032)
Supplement: Supplementary file 1 [file Table_1.docx]

Table S1. Characteristics of the relevant studies.

| Source | Study design | Subtype | Drug | No. of patients  NRAS mut/ wt | ORR (%)  NRAS mut/ wt | PFS (mos)  NRAS mut/ wt | OS (mos)  NRAS mut/ wt | Other Clinical outcomes, DCR (%), TTF (mos), et al  NRAS mut/ wt | Univariate and multivariate factors |
| --- | --- | --- | --- | --- | --- | --- | --- | --- | --- |
| Guida et al, 2021 (17) | Retrospective | Cutaneous; Unknown | Anti-PD-1  Ipilimumab  Anti-PD-1+ipilimumab | Total: 162/169 | 42 vs 37 | 12 vs 9 | 32 vs 27 | DCR: 60 vs 59 | Sex, age, NRAS status, site of primary melanoma, antiPD-1 vs ipilimumab, metastatic sites (<3 versus ≥3), WBC (+1000), Lymphocytes (+1000), N/L ratio (+1), Platelet (+100), LDH (<ULN versus >ULN), ECOG PS (2–3 versus 0–1) |
|  |  |  |  | Anti-PD-1: 114/132 | 43 vs 42 | 15 vs 16 | 26 vs 15 | DCR: 68 vs 67 |  |
| Kirchberger et al, 2018 (14) | Retrospective | NA | Anti-PD-1  Ipilimumab  Anti-PD-1+ipilimimab | Total: 128/236 | 21 vs 27 | NA | 33 vs 21 | DCR: 39 vs 56 | Gender, ECOG status, serum LDH, prior systemic therapy, Brain metastases, NRAS |
|  |  |  |  | Anti-PD-1: 34/8 | 21 vs 13 | NA | 18 vs 30 | DCR: 35 vs 25 |  |
| Douglas et al, 2015 (13) | Retrospective | acral (5%), mucosal (8%) | IL-2;  Ipilimumab;  Anti-PD-(L)1 | Total: 69/211 | 29 vs 17 | First-line:  4.1 vs 2.9 | First-line:  19.5 vs 15.2 | NA | Gender, age, stage, location of primary tumor, LDH, therapy, NRAS |
|  |  |  |  | Anti-PD-(L)1: 11/37 | 64 vs 30 |  |  |  |  |
| IMspire170,2020 (16) | RCT | Asian (2.7%) | Pembrolizumab | 104/119 | NA | 5.7 vs 4.1 | NA | NA | Sex, age, race, geographic region, ECOG PS at randomization, LDH status, PD-L1 status, disease status, metastatic disease stage, liver metastasis, brain metastasis, prior adjuvant therapy, prior adjuvant ipilimumab, NF1 mutation, NRAS |
| Joseph et al, 2012 (11) | Retrospective | NA | IL-2 | 15/88 | 47 vs 19 | 7.1 vs 2.3 | 63.6 vs 28.8  (17% stage IIIc) | NA | Age, sex, adjuvant IFN, prior systemic therapy for metastatic disease, LDH, stage, NRAS |
| Mangana et al, 2015 (12) | Retrospective | Acral (11%), mucosal (5%) | Ipilimumab | 62 (BRAF included) vs 39 | NA | NA | 12.1 vs 8.03 (BRAF mut) vs 8.26 | NA | BRAF or NRAS mutant |
| Shoushtari, et al, 2021 (18) | Retrospective | Cutaneous, unknown | Anti-PD-1, nivolumab+ipilimumab |  | NA | NA | NA | TTF of anti-PD-1 (NRAS vs BRAF V600 vs NF1 vs other): 4.2 vs 7.5 vs 22 vs NR | Driver, LDH, site, ECOG, age, sex, stage, neutrophil-lymphocyte ratio, relative eosinophil count, relative lymphocyte count |

RCT, randomized clinical trial, only patients received anti-PD(L)1 monotherapy involved; b. high-dose interleukin-2; ORR, objective response rate; PFS, progression-free survival; OS, overall survival; DCR, disease control rate; TTF, time to treatment failure; NA, not available; NR, not reached.
